# New products

**DOI:** 10.1155/S1463924600000377

**Published:** 2000


Journal of Automated Methods & Management in Chemistry, Vol. 22, No. 6 (November–December 2000) pp. 205–206

### New products

### Zymark New Pipetting Workstation with Plate-stacking

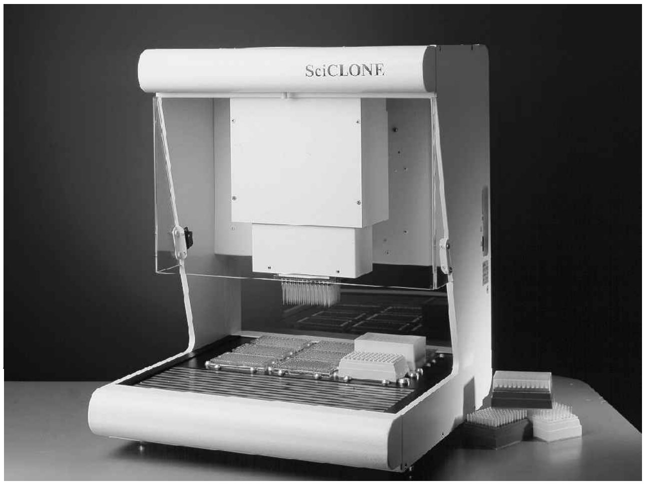

*The Scitec™ SciCLONE™ pipetting workstation.*

The Scitex™ SciCLONE™ pipetting workstation provides fast and accurate liquid transfers, reagent additions, and plate reformatting with 96-well and 384-well microplate formats. The SciCLONE contains an advanced *X–Y–Z* positioning capability that enables rapid positioning of the plates and pipetting head to accomplish its liquid handling operations without delay. The intregrated nine-position worktable accommodates any configuration of microplates, reagents, accessories or disposable pipette tips. The software allows easy modification of the parameters associated with the transfer operations, including transfer volumes, dispensing locations and tip touching.

The SciCLONE can be configured with Scitec's Auto-Stack System, a sophisticated storage device for housing microplates, deepwell plates, vials and pipette tips for use within an automated system. With capability for handling up to 100 independent shelves, the AutoStack provides extended capacity and walkaway time when performing automated pipetting applications.

Additional accessories for the SciCLONE include a disposable tip server system, an automated dispenser that can load and unload the SciCLONE desk positions with disposable tip cassettes, and a reagent dispenser module that provides bulk reagent dispensing directly into plates or reservoirs.

*For more information contact Lynda Thomas, Marketing Communications. Tel: +1 (508) 497 6549; e-mail: lynda.thomas@zymark.com.*

### Zymark Presto™ AutoStack™ increases plate storage capacity

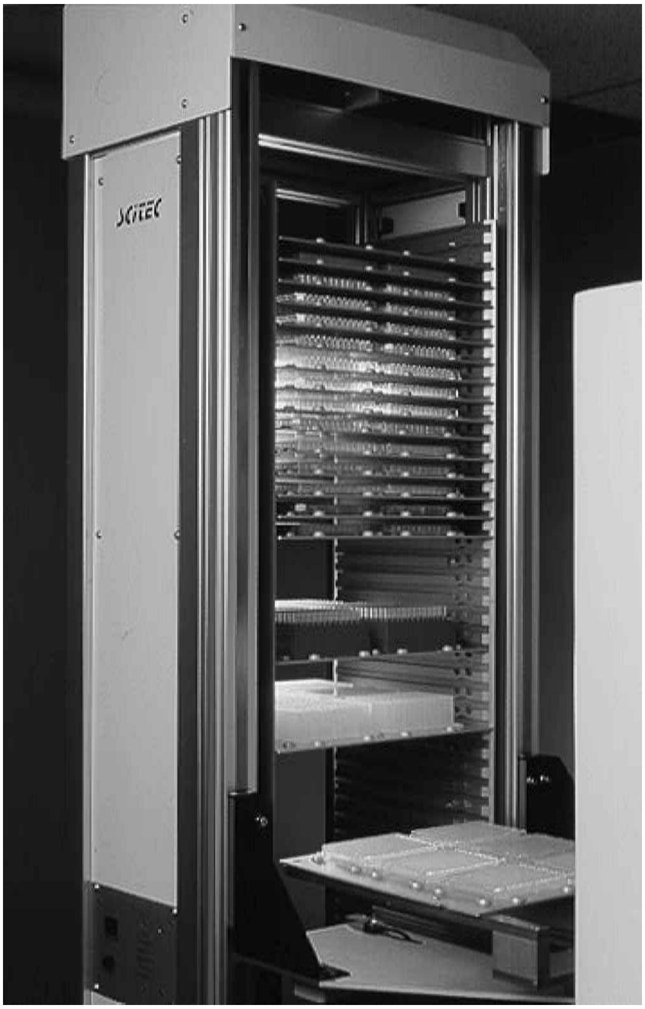

*The Zymark Presto™ AutoStack™.*

The new Zymark Presto AutoStack increases the microplate storage capacity and flexibility of the Tecan Genesis and other liquid handling workstations. Designed to provide fast, reliable, access to consumables used in 'typical' microplate assays, the AutoStack presents trays of the tips, reagents, microplates or deep wells to an automated device without robotic arm intervention. Using ActiveX software, the AutoStack can be integrated with a large variety of automation devices, including liquid handlers, automated gantry grippers and robotic arms that are used in storage and retrieval, cherry picking or other assays.

The AutoStack's shelf spacing and elevator access allows for dynamic re-allocating of its contents. It is available in a standard size of nine-plates-per-tray, 70-tray configuration for storage of up to 630 microplates. Optional capabilities include an Automatic Cover Removal that provides a means of working with covered microplates and an insulated enclosure for environmental control.

Zymark impacts the world by advancing the ability to discover, develop and market effective drugs that can lengthen and improve the quality of life for everyone. Zymark's employees are active in more than 35 countries and their combined expertise in Pharmaceutical Analysis and Laboratory Automation makes Zymark the world's leading supplier in this application area.

*For more information contact Lynda Thomas, Marketing Communications. Tel: +1 (508) 497 6549; e-mail: lynda.thomas@zymark.com.*

